# Implications for sequencing of biologic therapy and choice of second anti‐TNF in patients with inflammatory bowel disease: results from the IMmunogenicity to Second Anti‐TNF therapy (IMSAT) therapeutic drug monitoring study

**DOI:** 10.1111/apt.17170

**Published:** 2022-08-29

**Authors:** Neil Chanchlani, Simeng Lin, Marcus K. Auth, Chai Leng Lee, Helena Robbins, Shi Looi, Senthil V. Murugesan, Tom Riley, Cathryn Preston, Sophie Stephenson, Wendy Cardozo, Sunil A. Sonwalkar, Mohammed Allah‐Ditta, Lynne Mansfield, Dharmaraj Durai, Mark Baker, Ian London, Emily London, Sanjay Gupta, Alex Di Mambro, Aisling Murphy, Edward Gaynor, Kelsey D. J. Jones, Andrew Claridge, Shaji Sebastian, Sankaranarayanan Ramachandran, Christian P. Selinger, Simon P. Borg‐Bartolo, Paul Knight, Michael B. Sprakes, Julie Burton, Patricia Kane, Stephanie Lupton, Aimee Fletcher, Daniel R. Gaya, Roghan Colbert, John Paul Seenan, Jonathan MacDonald, Lucy Lynch, Iain McLachlan, Stephanie Shields, Richard Hansen, Lisa Gervais, Mwansa Jere, Muhammad Akhtar, Karen Black, Paul Henderson, Richard K. Russell, Charlie W. Lees, Lauranne A. A. P. Derikx, Melanie Lockett, Frederica Betteridge, Aminda De Silva, Arif Hussenbux, John Beckly, Oliver Bendall, James W. Hart, Amanda Thomas, Ben Hamilton, Claire Gordon, Desmond Chee, Timothy J. McDonald, Rachel Nice, Marian Parkinson, Helen Gardner‐Thorpe, Jeff R. Butterworth, Asima Javed, Sarah Al‐Shakhshir, Rekha Yadagiri, Sebrene Maher, Richard C. G. Pollok, Tze Ng, Priscilla Appiahene, Fiona Donovan, James Lok, Rajiv Chandy, Reema Jagdish, Daniyal Baig, Zahid Mahmood, Liane Marsh, Allison Moss, Amin Abdulgader, Angus Kitchin, Gareth J. Walker, Becky George, Yuen‐Hui Lim, James Gulliver, Stuart Bloom, Holly Theaker, Sean Carlson, J. R. Fraser Cummings, Robert Livingstone, Amanda Beale, Josiah O. Carter, Andrew Bell, Archibald Coulter, Jonathon Snook, Helen Stone, Nicholas A. Kennedy, James R. Goodhand, Tariq Ahmad, IMSAT study investigators

**Affiliations:** ^1^ Royal Devon University Healthcare NHS Foundation Trust Exeter UK; ^2^ Alder Hey Children's NHS Foundation Trust Liverpool UK; ^3^ Ashford and St Peter's Hospitals NHS Foundation Trust Chertsey UK; ^4^ Blackpool Teaching Hospitals NHS Foundation Trust Blackpool UK; ^5^ Bradford Teaching Hospitals NHS Foundation Trust Bradford UK; ^6^ Calderdale and Huddersfield Royal Infirmary Huddersfield UK; ^7^ Cardiff and Value University Health Board Cardiff UK; ^8^ Countess of Chester Hospital NHS Foundation Trust Chester UK; ^9^ Croydon Health Services NHS Trust Croydon UK; ^10^ Gloucestershire Hospitals NHS Foundation Trust Gloucester UK; ^11^ Great Ormond Street Hospital London UK; ^12^ Great Western Hospitals NHS Foundation Trust Swindon UK; ^13^ Hull University Teaching Hospitals NHS Trust Hull UK; ^14^ Leeds Teaching Hospitals NHS Trust Leeds UK; ^15^ Manchester University NHS Foundation Trust Manchester UK; ^16^ Mid Yorkshire Hospitals NHS Trust Wakefield UK; ^17^ NHS Greater Glasgow and Clyde – Glasgow Royal Infirmary Glasgow UK; ^18^ NHS Greater Glasgow and Clyde – Queen Elizabeth University Hospital Glasgow UK; ^19^ NHS Greater Glasgow and Clyde – Royal Hospital for Sick Children Glasgow UK; ^20^ NHS Lanarkshire ‐ University Hospital Wishaw Wishaw UK; ^21^ NHS Lothian ‐ Royal Hospital For Sick Children Edinburgh UK; ^22^ NHS Lothian ‐ Western General Hospital Edinburgh UK; ^23^ North Bristol NHS Trust Bristol UK; ^24^ Royal Berkshire NHS Foundation Trust Reading UK; ^25^ Royal Cornwall Hospitals NHS Trust Cornwall UK; ^26^ Shrewsbury and Telford Hospital NHS Trust Shrewsbury UK; ^27^ St George's University Hospitals NHS Foundation Trust London UK; ^28^ St Helens and Knowsley Teaching Hospitals NHS Trust Rainhill UK; ^29^ Stockport NHS Foundation Trust Stockport UK; ^30^ Taunton and Somerset NHS Foundation Trust Taunton UK; ^31^ Torbay and South Devon NHS Foundation Trust Torbay UK; ^32^ University College London Hospitals NHS Foundation Trust London UK; ^33^ University Hospital Southampton NHS Foundation Trust Southampton UK; ^34^ University Hospitals Bristol and Weston NHS Foundation Trust–Bristol Bristol UK; ^35^ University Hospitals Bristol and Weston NHS Foundation Trust–Weston Weston UK; ^36^ University Hospitals Dorset NHS Foundation Trust Poole UK

**Keywords:** adalimumab, antibodies, anti‐TNF, drug persistence, immunogenicity, infliximab, therapeutic drug monitoring, treatment failure

## Abstract

**Background:**

Anti‐drug antibodies are associated with treatment failure to anti‐TNF agents in patients with inflammatory bowel disease (IBD).

**Aim:**

To assess whether immunogenicity to a patient's first anti‐TNF agent would be associated with immunogenicity to the second, irrespective of drug sequence

**Methods:**

We conducted a UK‐wide, multicentre, retrospective cohort study to report rates of immunogenicity and treatment failure of second anti‐TNF therapies in 1058 patients with IBD who underwent therapeutic drug monitoring for both infliximab and adalimumab. The primary outcome was immunogenicity to the second anti‐TNF agent, defined at any timepoint as an anti‐TNF antibody concentration ≥9 AU/ml for infliximab and ≥6 AU/ml for adalimumab.

**Results:**

In patients treated with infliximab and then adalimumab, those who developed antibodies to infliximab were more likely to develop antibodies to adalimumab, than patients who did not develop antibodies to infliximab (OR 1.99, 95%CI 1.27–3.20, *p* = 0.002). Similarly, in patients treated with adalimumab and then infliximab, immunogenicity to adalimumab was associated with subsequent immunogenicity to infliximab (OR 2.63, 95%CI 1.46–4.80, *p* < 0.001). For each 10‐fold increase in anti‐infliximab and anti‐adalimumab antibody concentration, the odds of subsequently developing antibodies to adalimumab and infliximab increased by 1.73 (95% CI 1.38–2.17, *p* < 0.001) and 1.99 (95%CI 1.34–2.99, *p* < 0.001), respectively. Patients who developed immunogenicity with undetectable drug levels to infliximab were more likely to develop immunogenicity with undetectable drug levels to adalimumab (OR 2.37, 95% CI 1.39–4.19, *p* < 0.001). Commencing an immunomodulator at the time of switching to the second anti‐TNF was associated with improved drug persistence in patients with immunogenic, but not pharmacodynamic failure.

**Conclusion:**

Irrespective of drug sequence, immunogenicity to the first anti‐TNF agent was associated with immunogenicity to the second, which was mitigated by the introduction of an immunomodulator in patients with immunogenic, but not pharmacodynamic treatment failure.

## Graphical abstract

In 1058 patients with inflammatory bowel disease who underwent therapeutic drug monitoring for both infliximab and adalimumab, those who developed antibodies to a first anti‐TNF were more likely to develop antibodies to a second anti‐TNF, irrespective of drug sequence. Commencing an immunomodulator at the time of switching to the second anti‐TNF was associated with improved drug persistence in patients with immunogenic but not pharmacodynamic failure.

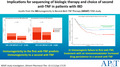

## 1 INTRODUCTION

The anti‐TNF monoclonal antibodies infliximab and adalimumab have transformed the management of immune‐mediated inflammatory diseases (IMIDs), including inflammatory bowel disease (IBD).^1^

Regrettably, however, anti‐TNF treatment failure is common. Obesity, cigarette smoking, higher baseline markers of disease activity, anti‐TNF monotherapy and the development of anti‐drug antibodies are associated with low drug levels and anti‐TNF treatment failure.^2^ Loss of response is frequently associated with low anti‐TNF drug levels and the formation of anti‐drug antibodies, which can be predicted by the carriage of the HLA‐DQA1*05 haplotype,^3^, ^4^ and mitigated by concomitant immunomodulator use.^2^

Whilst it is generally accepted that there is a diminishing return from second‐ and subsequent anti‐TNF therapies,^5^, ^6^ well‐designed and adequately powered sequencing studies are scarce.^7^, ^8^ Most have been small and limited to the immunogenicity of second‐line adalimumab because historically infliximab has been used first. Estimates range from 28 to 40%^7^, ^9^, ^10^, ^11^, ^12^ and 39 to 70%^7^, ^12^, ^13^ for the risk of immunogenicity to second‐line adalimumab and infliximab, respectively. Few studies have addressed whether the development of antibodies to the first anti‐TNF drug is associated with immunogenicity^8^, ^10^, ^11^, ^12^, ^13^, ^14^, ^15^ and treatment failure to a second.

The aim of the IMplications for Sequencing of biologic therapy and choice of second Anti‐TNF in patients with IBD (IMSAT) study was to evaluate the relationship between immunogenicity to the first anti‐TNF therapy and immunogenicity and drug persistence to second anti‐TNF therapy. We hypothesized that immunogenicity to the first anti‐TNF would be associated with immunogenicity to the second anti‐TNF, irrespective of drug sequence.

## 2 MATERIALS AND METHODS

### 2.1 Study design, clinical setting, and participants

We sought to define the:

1. Risk of immunogenicity to a second anti‐TNF drug, stratified by immunogenicity to the first anti‐TNF drug.
2. Rates of drug persistence to a second anti‐TNF, following treatment failure to the first anti‐TNF, stratified by immunogenicity to the first anti‐TNF drug.
3. Strategies to mitigate the development of immunogenicity to a second anti‐TNF drug.

We conducted a UK‐wide, multicentre, retrospective cohort study to report rates of immunogenicity to second anti‐TNF therapies in patients with IBD.

The Academic Department of Blood Sciences at the Royal Devon and Exeter NHS Foundation Trust provides a therapeutic drug monitoring (TDM) service to hospitals throughout the United Kingdom (UK).^16^ All patients who had drug and anti‐drug antibody levels undertaken for both infliximab and adalimumab, originator or biosimilar preparations, between 1 May 2013 and 31 November 2020 were eligible for inclusion. Sites who had sent samples for TDM measurement for >2 patients were invited to take part in our study.

Patient eligibility was confirmed by local research sites. We included patients with a diagnosis of Crohn's disease, ulcerative colitis (UC), and IBD‐unclassified (IBD‐U) as determined by local sites. Using case note reviews of secondary care records, their disease courses were followed to the point of data entry or drug withdrawal. Patients who had historically been treated with an anti‐TNF drug prior to the index course with TDM measurement, those who had not been exposed to two anti‐TNF drugs, and where the clinical data were incomplete were excluded.

### 2.2 Outcome measures

The primary outcome was immunogenicity to the second anti‐TNF drug, defined at any timepoint as an anti‐TNF antibody concentration ≥9 AU/ml for infliximab and ≥6 AU/ml for adalimumab, using the Immundiagnostik anti‐drug antibody ELISA.^16^ The secondary outcome was second anti‐TNF drug persistence, defined as the length of time from initiation of second anti‐TNF to discontinuation of therapy.^17^

Treatment failure endpoints were primary non‐response at week 20, loss of response after week 20 and adverse events leading to drug withdrawal:

*Primary non‐response*: was defined as exit before week 20 because of treatment failure (including resectional inflammatory bowel disease surgery), corticosteroid use at week 20 (new prescriptions or if the previous dose had not been stopped), or physician global assessment of no meaningful response at any time prior to drug withdrawal, even if the drug continues beyond standard induction period.

*Loss of response*: in patients who did not have primary non‐response was defined as symptomatic inflammatory bowel disease activity that warranted an escalation of steroid, immunomodulator or anti‐TNF therapy, resectional surgery, or exit from the study due to treatment failure.^2^ Timing of loss of response was defined as the time of treatment escalation, drug withdrawal or surgery.

*Adverse events* were coded centrally according to the Medical Dictionary for Regulatory Activities (MedDRA) version 23.1. Serious adverse events included those that required hospitalisation, were life‐threatening or resulted in persistent, permanent or substantial disability or incapacity. Causality was graded according to the Good Clinical Practice framework guidelines as not related, unlikely, possibly, probably or definitely related to treatment by local research sites.^18^

We subsequently incorporated the use of TDM‐based decision making in the setting of primary non‐response or loss of response, according to the results of their most recent drug level and anti‐drug antibodies to the first anti‐TNF.^2^, ^19^, ^20^, ^21^

1. Immunogenic—pharmacokinetic failure was defined as treatment failure with low anti‐TNF drug levels (infliximab <3 mg/L, adalimumab <5 mg/L), and the presence of anti‐TNF antibodies (infliximab ≥9 AU/mL, adalimumab ≥6 AU/ml).
2. Immunogenic—pharmacodynamic failure was defined as treatment failure despite adequate anti‐TNF drug levels (infliximab ≥3 mg/L, adalimumab ≥5 mg/L), and the presence of anti‐TNF antibodies (infliximab ≥9 AU/mL, adalimumab ≥6 AU/ml).
3. Non‐immunogenic—pharmacokinetic failure was defined as treatment failure with low anti‐TNF drug levels (infliximab <3 mg/L, adalimumab <5 mg/L), and without the presence of anti‐TNF antibodies (infliximab <9 AU/ml, adalimumab <6 AU/mL).
4. Non‐immunogenic—pharmacodynamic failure was defined as treatment failure despite adequate anti‐TNF drug levels (infliximab ≥3 mg/L, adalimumab ≥5 mg/L), and without the presence of anti‐TNF antibodies (infliximab <9 AU/ml, adalimumab <6 AU/ml).

Time to loss of response was defined as the duration of time from initiation of anti‐TNF therapy to treatment failure. Non‐treatment failure endpoints were withdrawal of anti‐TNF therapy in patients with quiescent disease, by treating physician or patient choice.

### 2.3 Variables

We recorded demographic (sex, age, ethnicity, weight, smoking history), IBD‐related data (date of diagnosis, phenotype) according to Montreal Classification, and immunomodulator status (type, dosing and frequency at the time of start and end of anti‐TNF treatment), with no minimum duration required and anti‐TNF treatment data (indication, dosing frequency, interval, reason for withdrawal, treatment plan after cessation and any breaks in treatment ≥16 weeks).

### 2.4 Laboratory methods

All laboratory analyses were performed at the Academic Department of Blood Sciences at the Royal Devon and Exeter NHS Foundation Trust. Anti‐TNF drug and anti‐drug antibodies were measured on the Dynex Technologies (Chantilly, Virginia, USA) DS2 automated ELISA platform.

The Immundiagnostik (IDK) AG (Bensheim, Germany) IDKmonitor infliximab (K9654) and adalimumab (K9651) total anti‐drug antibody assays allow semi‐quantitative measur‐drug antibodies.^22^, ^23^ A pre‐treatment acid dissociation step is used to separate anti‐drug antibodies from the therapeutic antibody. The assay then follows a standard ELISA format using a recombinant therapeutic antibody as a capture and detection antibody. The positivity threshold for anti‐infliximab antibodies is 9 AU/ml and for anti‐adalimumab antibodies is 6 AU/ml.^16^

The IDKmonitor free infliximab (K9655) and adalimumab (K9657) drug level assays permit quantitative measurement of a free therapeutic drug in serum.^22^, ^23^ The assays follow a standard ELISA format using a specific monoclonal anti‐drug antibody fragment as a capture antibody and a peroxidase‐labelled anti‐human IgG antibody as a detection antibody. The measuring range for both assays is 0.8–45 mg/L, with the absence of drug being defined using a cutoff of <0.8 mg/L.

### 2.5 Statistical analysis

At the time of study design, we identified approximately 1000 patients who had TDM results for both anti‐TNF drugs: 78% were treated with infliximab first, and 22% with adalimumab first. We assumed that the crude rates of immunogenicity according to biologic type were generalisable across the cohort and allowed for a 30% attrition rate. We calculated that our sample size provided 93% and 79% power at the 0.025 significance threshold level to detect a significant association between immunogenicity to the first and second anti‐TNF, in the infliximab‐ and adalimumab‐ treated first cohorts, respectively.

Data were pseudonymized and entered into a purpose‐designed electronic database in REDCap (Vanderbilt University Medical Centre, Tennessee, US).^24^ Statistical analyses were undertaken in R 4.0.4 (R Foundation for Statistical Computing, Vienna, Austria). All tests were two‐tailed and *p*‐values <0.05 were considered significant. We included patients with missing clinical data in analyses for which they had data and specified the denominator for each variable.

We performed univariable analyses using Fisher's exact and Mann–Whitney U tests to identify categorical and continuous variables associated with immunogenicity and treatment failure outcomes. Logistic regression analyses were used to assess whether the magnitude of anti‐drug antibodies to the first anti‐TNF was independently associated with antibody formation to the second anti‐TNF. We performed sensitivity analyses according to drug clearance, which was defined as undetectable anti‐TNF drug levels (infliximab <0.8 mg/L, adalimumab <0.8 mg/L), and the presence of anti‐TNF antibodies (infliximab ≥9 AU/ml, adalimumab ≥6 AU/ml).

Rates of drug persistence were estimated using the Kaplan–Meier method, and comparative analyses were performed using Cox proportional hazards regression. Patients were censored at the time of treatment failure to their second anti‐TNF.

Youden's formula^25^ was used to determine the optimal anti‐drug antibody titre during the first anti‐TNF therapy to predict immunogenicity with undetectable drug level to second anti‐TNF, and receiver operator characteristic curves and area under the curve analyses with bootstrapping were used to estimate the diagnostic accuracy of the model.

## 3 RESULTS

### 3.1 Patient identification and eligibility

**FIGURE 1:**
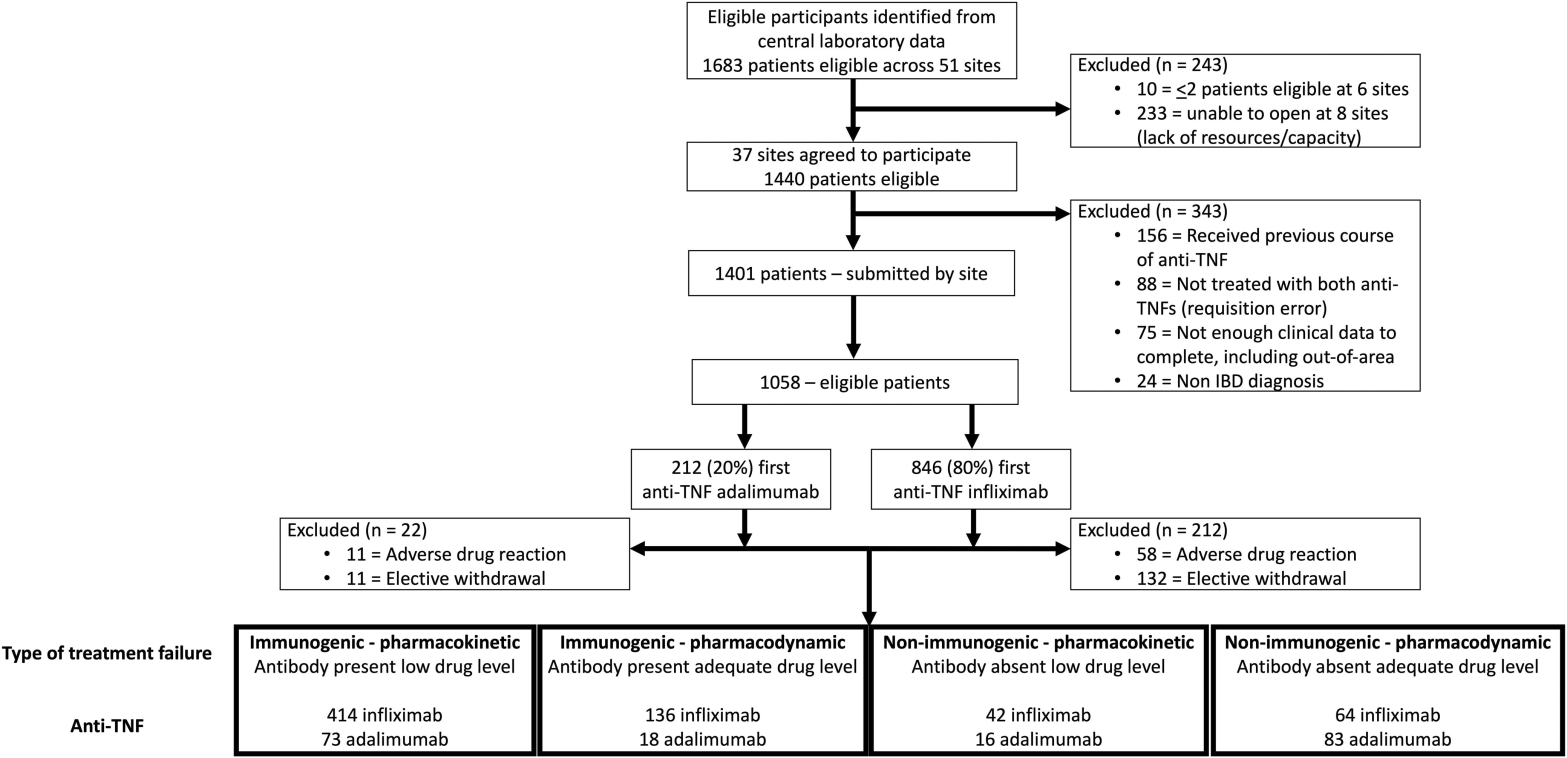
Patient disposition.

Between 1 May 2013 and 31 November 2020, we identified 38,940 and 14,847 TDM results, from 13,708 and 8662 patients, treated with infliximab (median 2, range [1–48]) and adalimumab (1, [1–17]), respectively. One thousand six hundred eighty‐three patients from 51 sites had both infliximab and adalimumab TDM results (Figure 1; Table S1). Six sites submitted ≤2 patients (*n* = 10 patients) so were not approached and eight sites (*n* = 233 patients) opted not to take part.

A total of 1440 patients were screened by research sites for eligibility, and data for 97.3% (1401/1440) patients were submitted. We excluded 11.1% (156/1401) patients who had received a previous course of anti‐TNF therapy; 6.3% (88/1401) patients where a requisition error had occurred and who had never received a second anti‐TNF; 5.4% (75/1401) patients with incomplete clinical data; and 1.7% (24/1401) patients who did not have IBD.

### 3.2 Patient characteristics

Of the 1058 (50.3% [532] male) patients in the final analysis: 71.4% (755), 24.4% (258), and 4.3% (45) patients were diagnosed with Crohn's disease, UC, and IBD‐U, respectively. The median time of follow‐up from starting the first anti‐TNF to the point of data entry or drug withdrawal was 3.84 years (IQR 2.34–5.68). 80.0% (846) patients were treated with infliximab and then adalimumab, and 20.0% (212) patients were treated with adalimumab and then infliximab. There was no difference in the duration of treatment with the first anti‐TNF drug (infliximab: 1.4 years [IQR 0.7–2.9], adalimumab: 1.3 [IQR 0.6–2.5], *p* = 0.179). The first anti‐TNF was discontinued in 80% (846/1058) patients because they did not respond or lost response; 6.6% (70/1058) patients developed an adverse event leading to drug cessation and the drug was withdrawn in 13.4% (142/1058) patients for non‐treatment failure reasons (physician recommendation: 78.2% [111/142], patient choice: 21.8% [31/142]).

**FIGURE 2:**
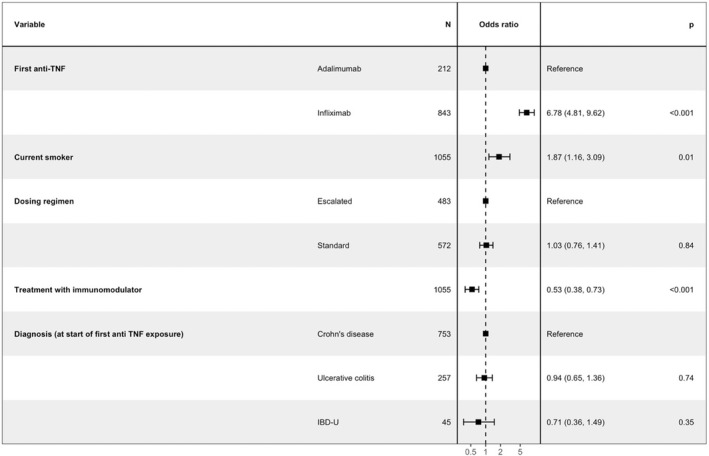
Forest plot showing the coefficients from a multivariable logistic regression model of associations with immunogenicity to first anti‐TNF.

Patient characteristics, stratified by the development of immunogenicity to their first anti‐TNF, are shown in Table 1; Tables S2 and S3. Multivariable logistic regression analyses confirmed that infliximab, compared with adalimumab, smoking, inflammatory disease (B1) in patients with Crohn's disease and anti‐TNF therapy without an immunomodulator, but not dosing regimen or diagnosis, were independently associated with the development of immunogenicity to first anti‐TNF (Figure 2; Figures S1 and S2).

**TABLE 1:** Variables associated with the development of immunogenicity to first anti‐TNF

		Immunogenicity to first anti‐TNF		
Variable	Level	Yes (*n* = 803)	No (*n* = 255)	*P*‐value
Gender	Male	76.7% (408/532)	23.3% (124/532)	0.566
	Female	75.1% (395/526)	24.9% (131/526)	
Age (years)	Start first anti‐TNF	29.2 (18.6–45.9)	29.5 (20.7–43.0)	0.659
	Paediatric (<18 years old)	82.3% (195/237)	17.7% (42/237)	0.010
Ethnicity	White: British	74.6% (647/867)	25.4% (220/867)	0.069
	Black: Caribbean	66.7% (4/6)	33.3% (2/6)	
	Asian: Indian	76.0% (19/25)	24.0% (6/25)	
Smoking	Current	83.0% (127/153)	17.0% (26/153)	0.025
Weight (kg)	Start first anti‐TNF	68.0 (55.0–80.2)	70.2 (60.0–85.1)	0.004
Disease	Crohn's disease	75.8% (572/755)	24.2% (183/755)	0.428
	Ulcerative colitis	77.5% (200/258)	22.5% (58/258)	
	IBD‐U	68.9% (31/45)	31.1% (14/45)	
Location	L1	72.3% (141/195)	27.7% (54/195)	0.214
	L2	78.8% (149/189)	21.2% (40/189)	
	L3	76.7% (277/361)	23.3% (84/361)	
	L4	55.6% (5/9)	44.4% (4/9)	
L4 modifier	True	74.2% (121/163)	25.8% (42/163)	0.534
Behaviour	B1	81.3% (370/455)	18.7% (85/455)	<0.001
	B2	69.2% (108/156)	30.8% (48/156)	
	B3	65.3% (94/144)	34.7% (50/144)	
Perianal disease	True	76.5% (179/234)	23.5% (55/234)	0.784
Extent	E1	80.8% (21/26)	19.2% (5/26)	0.365
	E2	72.1% (93/129)	27.9% (36/129)	
	E3	79.1% (117/148)	20.9% (31/148)	
First anti‐TNF	Infliximab	83.2% (704/846)	16.8% (142/846)	<0.001
	Adalimumab	46.7% (99/212)	53.3% (113/212)	
First anti‐TNF indication	Luminal disease	75.7% (771/1019)	24.3% (248/1019)	0.447
	Extraintestinal	77.4% (24/31)	22.6% (7/31)	1.000
	Co‐existing non‐IBD diagnosis	55.6% (10/18)	44.4% (8/18)	0.052
Immunomodulator	Start first anti‐TNF	73.3% (400/546)	26.7% (146/546)	0.044
Immunomodulator type	Azathioprine	72.1% (294/408)	27.9% (114/408)	0.551
	Mercaptopurine	73.3% (55/75)	26.7% (20/75)	
	Tioguanine	100.0% (2/2)	0.0% (0/2)	
	Methotrexate	80.0% (48/60)	20.0% (12/60)	
Duration (years)	First anti‐TNF	1.3 (0.7–2.6)	1.6 (0.7–3.4)	0.090
Dosing regimen^a^	Standard	75.4% (432/573)	24.6% (141/573)	0.718
	Escalated	76.5% (371/485)	23.5% (114/485)	
Treatment outcome	Treatment failure	75.8% (641/846)	24.2% (205/846)	0.288
	Adverse event	70.0% (49/70)	30.0% (21/70)	
	Non‐treatment failure	79.6% (113/142)	20.4% (29/142)	

^a^ Dosing regimen was defined as standard if for infliximab‐treated patients, treatment was 5 mg/kg, 8‐weekly, and for adalimumab‐treated patients, treatment was 40 mg, 2‐weekly. Escalated dosing regimen was defined as, for infliximab‐treated patients, an increase in dosing (for example,  ≥7.5 mg/kg) and/or shortening of interval (for example, ≤ 7‐weekly), and for adalimumab‐treated patients, an increase in dosing (for example, 80 mg) and/or shortening of interval (for example, 1‐weekly).

### 3.3 Immunogenicity to a second anti‐TNF drug

**FIGURE 3:**
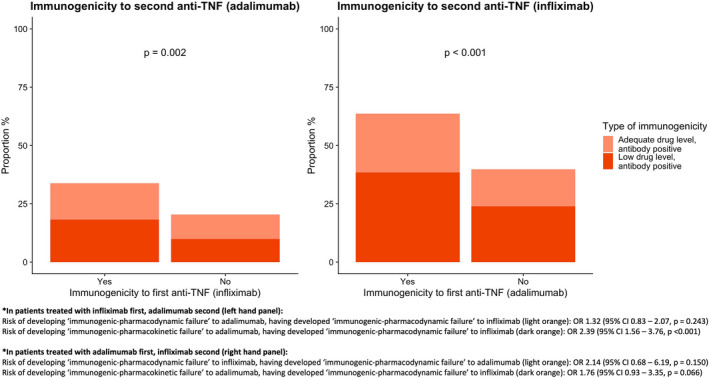
Risk of immunogenicity to second anti‐TNF, stratified by immunogenicity to first anti‐TNF.

In patients treated with infliximab then adalimumab, patients who developed antibodies to infliximab were more likely to develop antibodies to adalimumab, compared to patients who did not develop antibodies to infliximab (odds ratio [OR] 1.99, 95% confidence interval [CI] 1.27–3.20, *p* = 0.002) (Figure 3). Similarly, in patients treated with adalimumab and then infliximab, immunogenicity to adalimumab was associated with subsequent immunogenicity to infliximab (OR 2.63, 95% CI 1.46–4.80, *p* < 0.001).

For each 10‐fold increase in anti‐infliximab antibody concentration, the odds of subsequently developing antibodies to adalimumab increased by 1.73 (95% CI 1.38–2.17, *p* < 0.001). A similar observation was seen for patients who developed antibodies to adalimumab who were subsequently treated with infliximab (OR 1.99, 95%CI 1.34–2.99, *p* < 0.001).

Sensitivity analyses according to drug clearance (undetectable anti‐TNF drug levels, presence of antibodies) showed that patients who developed immunogenicity with undetectable drug levels to infliximab‐first were more than twice as likely to then develop immunogenicity with undetectable drug levels to adalimumab‐second (OR 2.37, 95% CI 1.39–4.19, *p* < 0.001). This was not seen for patients treated with adalimumab‐first and infliximab‐second (OR 1.85, 95% CI 0.88–3.87, *p* = 0.097) (Tables S4 and S5).

Youden's method demonstrated that the optimal anti‐drug antibody titre cut‐off to first anti‐TNF to determine immunogenicity with undetectable drug level to second anti‐TNF was 109 AU/mL for patients treated with infliximab first, with an area under the curve of 0.66 (95% CI 0.60–0.71). The sensitivity and specificity were 0.63 (95% CI 0.49–0.90) and 0.68 (95% CI 0.38–0.80), respectively. For patients treated with adalimumab first, the optimal anti‐drug antibody titre cut‐off was 11 AU/mL, with an area under the curve of 0.57 (95% CI 0.51–0.64). The sensitivity and specificity were 0.58 (95% CI 0.42–0.70) and 0.61 (95% CI 0.55–0.74), respectively.

### 3.4 Second anti‐TNF treatment outcomes

Overall, 39.3% (416/1058) patients did not respond or lost response to the second anti‐TNF, 4.3% (45/1058) patients developed an adverse drug reaction leading to drug cessation, and the drug was withdrawn electively in 4.3% (45/1058) patients.

Of the 846 patients who did not respond or who lost response to the first anti‐TNF, 57.6% (487/846) and 18.2% (154/846) patients were classified with immunogenic‐pharmacokinetic and immunogenic‐pharmacodynamic failure, respectively. A further 6.9% (58/846) and 17.4% (147/846) patients were classified with nonimmunogenic‐pharmacokinetic failure and nonimmunogenic‐pharmacodynamic failure, respectively (Table 2).

**TABLE 2:** Variables associated with treatment failure to first anti‐TNF, stratified by anti‐TNF therapy and type of treatment failure to first anti‐TNF

Infliximab as first anti‐TNF						
Treatment failure to first anti‐TNF	N	Immunomodulator status at the start of infliximab Proportion (95% CI)	Antibody level^a^ (IQR)	Drug level^b^ (IQR)	Escalated dosing regimen^c^ Proportion (95% CI)	Duration treated with infliximab [years (IQR)]
Immunogenic—pharmacokinetic Antibody present, low drug level	414	49.4% (95% CI 44.5–54.3)	102.0 (42.0–336.8)	<0.8	45.7% (95% CI 40.8–50.6)	1.2 (0.7–2.6)
Immunogenic—pharmacodynamic Antibody present, adequate drug level	136	53.7% (95% CI 44.9–62.2)	45.5 (16.0–72.8)	5.5 (4.0–9.1)	55.1% (95% CI 46.4–63.6)	1.9 (0.9–3.2)
Non‐immunogenic—pharmacokinetic Antibody absent, low drug level	42	69.0% (95% CI 52.8–81.9)	5.0 (5.0–5.0)	2.0 (0.7–2.7)	45.2% (95% CI 30.2–61.2)	1.8 (0.9–4.3)
Non‐immunogenic—pharmacodynamic Antibody absent, adequate drug level	64	70.3% (95% CI 57.4–80.8)	5.0 (5.0–5.0)	7.1 (4.4–13.9)	56.2% (95% CI 43.3–68.4)	2.1 (0.8–4.1)
**Adalimumab as first anti‐TNF**						
Treatment failure to first anti‐TNF	N	Immunomodulator status at the start of adalimumab Proportion (95% CI)	Antibody level^a^ (IQR)	Drug level^b^ (IQR)	Escalated dosing regimen^c^ Proportion (95% CI)	Duration treated with adalimumab [years (IQR)]
Immunogenic—pharmacokinetic Antibody present, low drug level	73	29.2% (95% CI 19.3–41.2)	201.0 (98.0–201.0)	<0.8	31.5% (95% CI 21.4–43.6)	1.4 (0.7–2.5)
Immunogenic—pharmacodynamic Antibody present, adequate drug level	18	55.6% (95% CI 31.3–77.6)	15.5 (11.2–62.8)	6.5 (5.9–11.4)	44.4% (95% CI 22.4–68.7)	1.4 (0.9–2.4)
Non‐immunogenic—pharmacokinetic Antibody absent, low drug level	16	43.8% (95% CI 20.8–69.4)	5.0 (5.0–5.0)	4.2 (1.8–4.4)	37.5% (95% CI 16.3–64.1)	0.7 (0.5–1.8)
Non‐immunogenic—pharmacodynamic Antibody absent, adequate drug level	83	44.6% (95% CI 33.8–55.9)	5.0 (5.0–5.0)	9.8 (8.2–13.7)	44.6% (95% CI 33.8–55.9)	1.5 (0.7–2.9)

^a^ Threshold for the presence of anti‐TNF antibodies: infliximab ≥9 AU/ml and adalimumab ≥6 AU/ml.

^b^ Threshold for adequate anti‐TNF drug level: infliximab ≥3 mg/L and adalimumab ≥7 mg/L.

^c^ Dosing regimen was defined as standard if for infliximab‐treated patients, treatment was 5 mg/kg, 8‐weekly, and for adalimumab‐treated patients, treatment was 40 mg, 2‐weekly. Escalated dosing regimen was defined as, for infliximab‐treated patients, an increase in dosing (for example, ≥ 7.5 mg/kg) and/or shortening of interval (for example, ≤ 7‐weekly), and for adalimumab‐treated patients, an increase in dosing (for example, 80 mg) and/or shortening of interval (for example, 1‐weekly).

The median duration of first anti‐TNF treatment was similar between patients treated with infliximab as first anti‐TNF and patients treated with adalimumab as first anti‐TNF (infliximab: 1.3 years [IQR 0.6–2.7] vs adalimumab: 1.4 years [IQR 0.6–2.6], *p* = 0.564), however, more patients treated with infliximab as first anti‐TNF were treated with a concomitant immunomodulator (infliximab: 53.4% [364/683] vs adalimumab: 40.5% [77/190], *p* = 0.002). Similar proportions of infliximab‐ and adalimumab‐treated patients had their first anti‐TNF dose escalated before switching drugs.

### 3.5 Second anti‐TNF drug persistence

**FIGURE 4:**
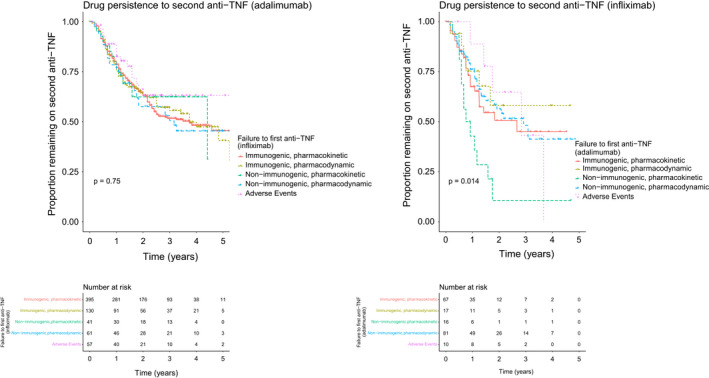
Drug persistence to second anti‐TNF, stratified by first anti‐TNF and type of failure to first anti‐TNF.

At 4‐year follow‐up, patients treated with adalimumab as a second anti‐TNF were more likely to continue the anti‐TNF therapy compared to patients treated with infliximab as a second anti‐TNF (adalimumab: 49.2% [95% CI 44.6–54.2] vs. infliximab: 37.8% [95% CI 28.8–49.6], *p* = 0.005). No differences were seen in drug persistence in patients treated with adalimumab as a second anti‐TNF, according to infliximab treatment failure status (Figure 4). In patients treated with infliximab as a second anti‐TNF, patients who developed non‐immunogenic‐pharmacokinetic failure had lower drug persistence compared to all other treatment failure groups. Sensitivity analyses demonstrated no difference in drug persistence to second‐anti TNF when applying a stricter definition of immunogenic, pharmacokinetic failure of undetectable drug level in the presence of antibodies (Figures S3 and S4).

### 3.6 Impact of immunomodulator on drug persistence

**FIGURE 5:**
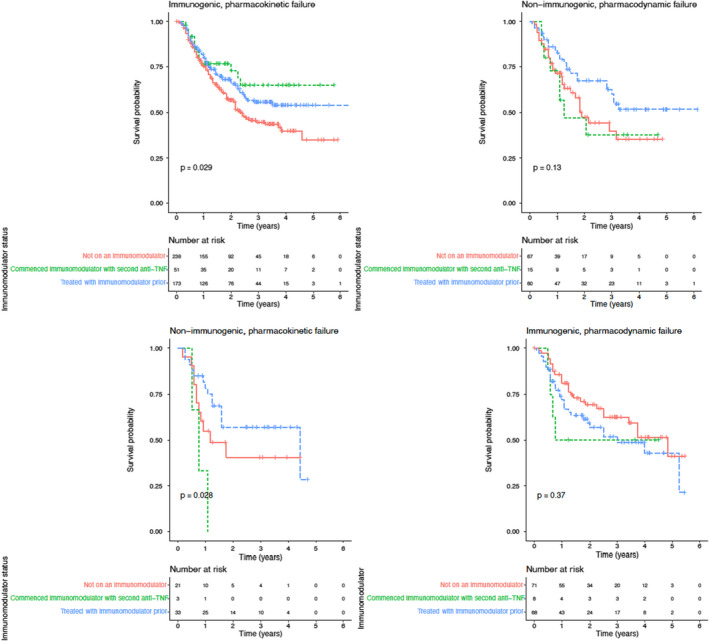
Drug persistence to second anti‐TNF, stratified by treatment failure to first anti‐TNF and immunomodulator status with second anti‐TNF.

Of patients who developed immunogenic, pharmacokinetic failure to their first anti‐TNF, those who commenced an immunomodulator with the second anti‐TNF, and those who were treated with an immunomodulator prior to starting second anti‐TNF, experienced longer drug persistence than patients who were not treated with an immunomodulator at time of second anti‐TNF (*p* = 0.029) (Figure 5). There was no difference in drug persistence in patients who commenced an immunomodulator at the time of second anti‐TNF or those who were treated with an immunomodulator prior to starting a second anti‐TNF (p = 0.355). No other associations between type of treatment failure to first anti‐TNF and immunomodulator status were observed.

### 3.7 Adverse events

Patients who developed an adverse event had lower drug persistence to first anti‐TNF than patients who developed treatment failure or non‐treatment failure (adverse event: 0.6 years [0.3–1.2], treatment failure: 1.5 [0.7–2.8], non‐treatment failure: 1.4 years [0.8–3.0]).

Adverse events leading to withdrawal of the second anti‐TNF occurred in 5.7% (12/212; 95% CI 3.1%–9.9%) patients treated with infliximab and 3.9% (33/846; 95% CI 2.7%–5.5%) patients treated with adalimumab. The most common adverse events were infusion and injection‐site reactions (52.2%, 60/115), rash (23.5%, 27/115), arthritis (3.5%, 4/115), and viral infections (3.5%, 4/115) (Table S6).

Infusion reactions to infliximab, which occurred after a median of 20.4 weeks (IQR 14.4–58.5), were associated with subsequent injection site reactions to adalimumab, which occurred after a median of 30.5 weeks (IQR 6.8–49.2) [69.0% (40/58) vs. 18.2% (6/33) *p* < 0.001]. Overall, infusion reactions to infliximab were associated with higher anti‐infliximab antibody levels; for every 10‐fold increase in antibodies, there was an 8 times risk of having an infusion reaction (OR 8.57, 95% CI 4.38–18.73, *p* < 0.001). No association was seen between injection site reactions and anti‐adalimumab antibody levels.

## 4 DISCUSSION

Irrespective of the anti‐TNF sequence, immunogenicity to the first anti‐TNF, was associated with immunogenicity to the second anti‐TNF. We report here that 34% (95% CI 30%–37%) and 64% (95% CI 54%–73%) of patients subsequently developed anti‐drug antibodies to adalimumab and infliximab, respectively. Patients who developed immunogenicity with undetectable drug levels to infliximab were more than twice as likely to develop immunogenicity with undetectable drug levels to adalimumab. Commencing an immunomodulator at the time of switching to the second anti‐TNF was associated with improved drug persistence in patients with immunogenic‐, but not pharmacodynamic‐treatment failure to the first anti‐TNF.

It is widely accepted that infliximab is more immunogenic than adalimumab. This has been attributed to the chimeric formulation of infliximab and the more variable drug levels and associated discontinuity of immune responses seen across the standard 8‐week dosing interval.^2^, ^26^

Why some individuals have a propensity to develop antibodies to unrelated epitopes of infliximab and then adalimumab is unknown.^27^ However, the dose‐effect observed here between the magnitude of antibody responses to the first anti‐TNF and the risk of developing antibodies to the second suggests that this association is not spurious.

We, like others, have shown previously that carriage of one or more HLA‐DQA1*05 alleles confers an almost two‐fold increased risk of immunogenicity to both infliximab and adalimumab, irrespective of concomitant immunomodulator use.^3^, ^4^ It is plausible then that some of the risks of sequential immunogenicity is explained by HLA‐DQA1*05 carriage. We were unable to replicate the association reported by Casteele et al,^7^ showing an association between drug level at the time of switch and subsequent immunogenicity to the second anti‐TNF. Our data argue against a mechanism common to both drugs accelerating clearance leading to subsequent immunogenicity. It is also possible that there is cross‐reactivity between both antibody assays and unmeasured antibodies such as hinge autoantibodies, rheumatoid factor, human anti‐mouse‐ or human anti‐human antibodies.^28^, ^29^, ^30^

We have replicated findings of a recent open‐label randomised controlled trial (RCT) that demonstrated reduced clinical failure rates in 90 patients with immunogenic‐pharmacokinetic treatment failure to first anti‐TNF who commenced azathioprine at the time of the switch to a second anti‐TNF.^8^ In our real‐world cohort of 1058 patients, 20% of whom were treated with adalimumab where immunogenicity rates are lower than for infliximab‐treated patients, we were powered to demonstrate the predictive risk of immunogenicity to patients treated with infliximab second‐line. Unlike the RCT performed by Roblin X et al. which only included patients who had immunogenic‐pharmacokinetic treatment failure, we were also able to demonstrate no additional benefit of an immunomodulator in patients who had pharmacodynamic failure to their first anti‐TNF, including in those who developed anti‐drug antibodies in the presence of adequate drug levels.

Herein, at the time of the first anti‐TNF treatment failure about one in five patients had anti‐drug antibodies that were detectable in the presence of the drug.^2^, ^31^, ^32^ Considerable uncertainty remains as to the function and relevance of these antibodies. Theoretically, they maybe neutralising, transient or maturing, and in the future may lead to a more robust immune response, and clear drug.^26^ Against them being clinically relevant, however, we found no association with subsequent immunogenicity, drug level, or the duration of treatment with the second anti‐TNF drug. Functional studies are required to better characterise these antibodies and to understand if they are clinically relevant.

As commonly performed in clinical practice, we incorporated the use of TDM‐based decision making in the setting of primary non‐response or loss of response. Consistent with recently published systematic reviews,^33^, ^34^ we stratified patients into one of four categories based on the presence or absence of antibodies and anti‐TNF drug concentration. Low anti‐TNF drug level cut‐offs were chosen based on the best available randomised controlled trial, prospective or post hoc analyses data that were associated with non‐remission. During maintenance therapy, for infliximab, based on randomised controlled trial data,^21^ this was determined to be 3 mg/L, and for adalimumab, based on the DIAMOND trial,^20^ this was determined to be 5 mg/L.

We acknowledge, however, the following limitations. First, inherent to our retrospective study design, we have no data on patients who failed an anti‐TNF drug but did not have TDM undertaken. Because of this we may have underestimated the rates of immunogenicity and overestimated drug persistence. This, and the lack of alternative biologic treatments during the timeframe of the study, probably accounts for why over half of patients, regardless of their immunogenicity status, were being treated with their second anti‐TNF after 4 years. Second, our results are potentially subject to interpretation bias, and bias because of missing data, including anti‐TNF and immunomodulator dose optimization data.

Third, we accept that our data would have been strengthened by objective markers of disease activity and endoscopic outcomes. Fourth, this was an unselected TDM referral cohort and although we recommend blood sampling just before the next dose, inevitably, some non‐trough samples will have been processed. Even drug‐tolerant anti‐drug antibody assays are not completely drug‐tolerant and therefore we are likely to have underestimated the rates of immunogenicity.^35^ This effect may be more important in adalimumab‐treated patients where TDM testing is more often ad‐hoc rather than immediately before administration as for infliximab.

Finally, although we were able to show that patients who developed immunogenicity with low drug levels to infliximab also developed this outcome to subsequent adalimumab because only 20% of our cohort were treated with adalimumab first, we were probably underpowered to demonstrate this association for patients treated with second‐line infliximab.

We collected data from multiple sites across the UK, who, based on the variability in the number of tests per patient, used a range of TDM practices. However, because we were able to confirm associations with immunogenicity that we reported in the prospective UK‐wide PANTS study,^2^ it is likely that our immunogenicity findings will be generalizable to other western populations. Whether sequential immunogenicity occurs in populations with low HLA‐DQA1*05 carriage and lower rates of immunogenicity is unknown.^4^, ^20^ Further research is needed to elucidate if patients who develop immunogenicity to one or more anti‐TNF drugs are also at risk of developing anti‐drug antibodies to the newer biologic therapies.

## 5 CONCLUSION

Patients who developed antibodies to their first anti‐TNF were more likely to develop antibodies to their second anti‐TNF, irrespective of drug sequence. Our findings support international recommendations for the management of anti‐TNF treatment failure, to switch out of biologic class when drug levels are therapeutic, and within class with an immunomodulator when anti‐TNF drug levels are low and associated with antibody development.

## AUTHOR CONTRIBUTIONS

Neil Chanchlani: conceptualization (equal); data curation (equal); formal analysis (equal); investigation (equal); methodology (equal); writing –original draft (equal); writing –review and editing (equal). Simeng Lin: data curation (equal); formal analysis (equal); investigation (equal). Rachel Nice: Data curation (equal); investigation (equal). Timothy J McDonald: Data curation (equal). Nicholas Alexander Kennedy: Data curation (equal); formal analysis (equal); investigation (equal); methodology (equal), supervision (equal). James R Goodhand: data curation (equal); investigation (equal); methodology (equal); supervision (equal); writing – review and editing (equal).Tariq Ahmad: Conceptualization (equal); data curation (equal); funding acquisition (equal); investigation (equal); methodology (equal); supervision (equal); writing – review and editing (equal); IMSAT study investigators: data curation (equal); investigation (equal).

## FUNDING INFORMATION

The study was funded by unrestricted grants from Janssen Pharmaceuticals and Cure Crohn's Colitis (Scottish‐registered charity). The funders had no role in the study design, data collection, data analysis, data interpretation or writing of the report. The corresponding author had full access to all the data in the study and had final responsibility for the decision to submit for publication.

## CONFLICT OF INTEREST

Neil Chanchlani has nothing to declare. Simeng Lin reports non‐financial support from Pfizer and Ferring, outside the submitted work. Nicholas A Kennedy reports grants from F. Hoffmann‐La Roche AG, grants from Biogen Inc, grants from Celltrion Healthcare, grants from Galapagos NV and non‐financial support from Immundiagnostik, during the conduct of the study; grants and non‐financial support from AbbVie, grants and personal fees from Celltrion, personal fees and non‐financial support from Janssen, personal fees from Takeda, personal fees and non‐financial support from Dr Falk, outside the submitted work. James R Goodhand reports grants from F. Hoffmann‐La Roche AG, grants from Biogen Inc, grants from Celltrion Healthcare, grants from Galapagos NV and non‐financial support from Immundiagnostik, during the conduct of the study. Tariq Ahmad reports grants and non‐financial support from F. Hoffmann‐La Roche AG, grants from Biogen Inc, grants from Celltrion Healthcare, grants from Galapagos NV and non‐financial support from Immundiagnostik, during the conduct of the study; personal fees from Biogen inc, grants and personal fees from Celltrion Healthcare, personal fees and non‐financial support from Immundiagnostik, personal fees from Takeda, personal fees from ARENA, personal fees from Gilead, personal fees from Adcock Ingram Healthcare, personal fees from Pfizer, personal fees from Genentech, non‐financial support from Tillotts, outside the submitted work.

## ETHICS STATEMENT

The choice of treatment, care or services was that of the healthcare professional and patient. In line with Health Research Authority guidelines, formal ethics approval for our study and patient consent were not required. The sponsor of the study is the Royal Devon and Exeter NHS Foundation Trust.

## Supporting information


**Appendix S1** Supporting Information

## Data Availability

The data will be made available to investigators whose proposed use of the data has been approved by an independent review committee. Analyses will be restricted to the aims in the approved proposal. Proposals should be directed to tariq.ahmad1@nhs.net. To gain access data requestors will need to sign a data access agreement.
