# Supplementary material for: Implications for sequencing of biologic therapy and choice of second anti‐TNF in patients with inflammatory bowel disease: results from the IMmunogenicity to Second Anti‐TNF therapy (IMSAT) therapeutic drug monitoring study
Source: Aliment Pharmacol Ther. 2022 Aug 29;56(8):1250–63. doi: 10.1111/apt.17170 (PMC9804266; doi:10.1111/apt.17170)
Supplement: Supplementary file 1 — Appendix S1 Supporting Information [file APT-56-1250-s001.docx]

**Supplemental file** for “Implications for sequencing of biologic therapy and choice of second anti-TNF in patients with inflammatory bowel disease: results from the Immunogenicity to Second Anti-TNF Therapy (IMSAT) therapeutic drug monitoring study”

Table of Contents

[**Supplementary Table 1:** IMSAT study investigators banner 2](#_Toc108717750)

[**Supplemental Table 2:** Variables associated with development of immunogenicity to first anti-TNF, stratified by biologic drug (infliximab as first anti-TNF) 7](#_Toc108717751)

[**Supplemental Table 3:** Variables associated with development of immunogenicity to first anti-TNF, stratified by biologic drug (adalimumab as first anti-TNF) 8](#_Toc108717752)

[**Supplemental Table 4:** Antibody concentration and drug level profiles, of patients who developed immunogenic-pharmacokinetic and immunogenic-pharmacodynamic failure to first anti-TNF, stratified by anti-TNF 9](#_Toc108717753)

[**Supplemental Table 5:** Risk of developing immunogenicity and immunogenic-pharmacokinetic failure (undetectable drug level) to second anti-TNF, stratified by condition 10](#_Toc108717754)

[**Supplemental Table 6:** Adverse Events leading to drug cessation 11](#_Toc108717755)

[**Supplemental Table 7:** Therapies following cessation to second anti-TNF 12](#_Toc108717756)

[**Supplemental Figure 1:** Forest plot showing the coefficients from a multivariable logistic regression model of associations with immunogenicity first anti-TNF, stratified by condition (Crohn’s disease) 13](#_Toc108717757)

[**Supplemental Figure 2:** Forest plot showing the coefficients from a multivariable logistic regression model of associations with immunogenicity first anti-TNF, stratified by condition (ulcerative colitis) 14](#_Toc108717758)

[**Supplemental Figure 3:** Drug persistence to second anti-TNF, including stricter definition of immunogenic, pharmacokinetic failure (antibody present, undetectable drug level), stratified by first anti-TNF and type of failure to first anti-TNF 15](#_Toc108717759)

[**Supplemental Figure 4:** Drug persistence to second anti-TNF, comparing patients who developed immunogenic-pharmacokinetic failure (antibody present, undetectable drug level) to patients who developed treatment failure for other reasons 16](#_Toc108717760)

### **Supplementary Table 1:** IMSAT study investigators banner

**1) First two authors on banner:**
Neil Chanchlani^1^ (first author, principle investigator)
Simeng Lin^1^ (second author)

**2) Collaborator list**

**3) Last three authors on banner:**
Nicholas A Kennedy^1^#
James R Goodhand^1^#
Tariq Ahmad^1^# (overall guarantor, chief investigator)
# denotes shared authorship

^1^Royal Devon University Healthcare NHS Foundation Trust, Exeter, UK

**Collaborator list**
* denotes principal investigator at collaborating site

| **Trust name** | **First name** | **Middle Initial** | **Surname** | **Title** | **Patients recruited** | | |
| --- | --- | --- | --- | --- | --- | --- | --- |
| Alder Hey Children’s NHS Foundation Trust | | | | | 33 | | |
|  | *Marcus | K | Auth | Consultant paediatric gastroenterologist |  |  |  |
|  | Chai Leng |  | Lee | Specialist registrar in paediatric gastroenterology |  | |  |
| Ashford and St Peter's Hospitals NHS Foundation Trust | | | | | 89 | | |
|  | *Helena |  | Robbins | Consultant gastroenterologist |  | |  |
|  | Shi |  | Looi | Specialist registrar in gastroenterology |  |  |  |
| Blackpool Teaching Hospitals NHS Foundation Trust | | | | | 18 | | |
|  | *Senthil | V | Murugesan | Consultant gastroenterologist |  | |  |
|  | Tom |  | Riley | Specialist registrar in gastroenterology |  |  |  |
| Bradford Teaching Hospitals NHS Foundation Trust | | | | | 8 | | |
|  | *Cathryn |  | Preston | Consultant gastroenterologist |  | |  |
|  | Sophie |  | Stephenson | Gastroenterology research nurse |  |  |  |
|  | Wendy |  | Cardozo | Gastroenterology research nurse |  |  |  |
| Calderdale and Huddersfield Royal Infirmary | | | | | 21 | | |
|  | *Sunil | A | Sonwalkar | Consultant gastroenterologist |  | |  |
|  | Mohammed |  | Allah-Ditta | Specialist pharmacist in gastroenterology |  |  |  |
|  | Lynne |  | Mansfield | IBD clinical nurse specialist |  |  |  |
| Cardiff and Value University Health Board | | | | | 32 | | |
|  | *Dharmaraj |  | Durai | Consultant gastroenterologist |  | |  |
|  | Mark |  | Baker | Research nurse |  |  |  |
| Countess of Chester Hospital NHS Foundation Trust | | | | | 55 | | |
|  | *Ian |  | London | Consultant gastroenterologist |  | |  |
|  | Emily |  | London | Clinical research fellow |  |  |  |
| Croydon Health Services NHS Trust | | | | | 14 | | |
|  | *Sanjay |  | Gupta | Consultant gastroenterologist |  | |  |
| Gloucestershire Hospitals NHS Foundation Trust | | | | | 19 | | |
|  | *Alex |  | Di Mambro | Consultant gastroenterologist |  | |  |
|  | Aisling |  | Murphy | Clinical fellow in gastroenterology |  |  |  |
| Great Ormond Street Hospital | | | | | 33 | | |
|  | *Edward |  | Gaynor | Consultant paediatric gastroenterologist |  | |  |
|  | Kelsey | DJ | Jones | Consultant paediatric gastroenterologist |  |  |  |
| Great Western Hospitals NHS Foundation Trust | | | | | 12 | | |
|  | *Andrew |  | Claridge | Consultant gastroenterologist |  | |  |
| Hull University Teaching Hospitals NHS Trust | | | | | 77 | | |
|  | *Shaji |  | Sebastian | Professor of gastroenterology |  | |  |
|  | Sankaranarayanan |  | Ramachandran | Specialty doctor in gastroenterology |  |  |  |
| Leeds Teaching Hospitals NHS Trust | | | | | 24 | | |
|  | *Christian | P | Selinger | Consultant gastroenterologist |  | |  |
| Manchester University NHS Foundation Trust | | | | | 13 | | |
|  | *Simon | P | Borg-Bartolo | Consultant gastroenterologist |  | |  |
|  | Paul |  | Knight | Consultant gastroenterologist |  |  |  |
| Mid Yorkshire Hospitals NHS Trust | | | | | 39 | | |
|  | *Michael | B | Sprakes | Consultant gastroenterologist |  | |  |
|  | Julie |  | Burton | Research nurse |  |  |  |
|  | Patricia |  | Kane | Research nurse |  |  |  |
|  | Stephanie |  | Lupton | Research nurse |  |  |  |
|  | Aimee |  | Fletcher | Clinical trials assistant |  |  |  |
| NHS Greater Glasgow and Clyde – Glasgow Royal Infirmary | | | | | 35 | | |
|  | *Daniel | R | Gaya | Consultant gastroenterologist |  | |  |
|  | Roghan |  | Colbert | Senior clinical fellow in gastroenterology |  |  |  |
| NHS Greater Glasgow and Clyde – Queen Elizabeth University Hospital | | | | | 60 | | |
|  | *John Paul |  | Seenan | Consultant gastroenterologist |  | |  |
|  | Jonathan |  | MacDonald | Consultant gastroenterologist |  |  |  |
|  | Lucy |  | Lynch | Specialist registrar in gastroenterology |  |  |  |
|  | Iain |  | McLachlan | Clinical development fellow |  |  |  |
|  | Stephanie |  | Shields | Clinical research fellow |  |  |  |
| NHS Greater Glasgow and Clyde – Royal Hospital for Sick Children | | | | | 10 | | |
|  | *Richard |  | Hansen | Consultant paediatric gastroenterologist |  | |  |
|  | Lisa |  | Gervais | Inflammatory bowel disease/research nurse specialist |  |  |  |
|  | Mwansa |  | Jere | Clinical research fellow |  |  |  |
| NHS Lanarkshire - University Hospital Wishaw | | | | | 4 | | |
|  | *Muhammad |  | Akhtar | Consultant gastroenterologist |  | |  |
|  | Karen |  | Black | Senior research nurse |  |  |  |
| NHS Lothian - Royal Hospital For Sick Children | | | | | 5 | | |
|  | *Paul |  | Henderson | Consultant paediatric gastroenterologist |  | |  |
|  | Richard | K | Russell | Professor of paediatric gastroenterology |  |  |  |
| NHS Lothian - Western General Hospital | | | | | 47 | | |
|  | *Charlie | W | Lees | Professor of gastroenterology |  | |  |
|  | Lauranne | AAP | Derikx | Senior clinical fellow in gastroenterology |  |  |  |
| North Bristol NHS Trust | | | | | 43 | | |
|  | *Melanie |  | Lockett | Consultant gastroenterologist |  | |  |
|  | Frederica |  | Betteridge | Specialist registrar in gastroenterology |  |  |  |
| Royal Berkshire NHS Foundation Trust | | | | | 14 | | |
|  | *Aminda |  | De Silva | Consultant gastroenterologist |  | |  |
|  | Arif |  | Hussenbux | Specialist registrar in gastroenterology |  |  |  |
| Royal Cornwall Hospitals NHS Trust | | | | | 51 | | |
|  | *John |  | Beckly | Consultant gastroenterologist |  | |  |
|  | Oliver |  | Bendall | Specialist registrar in gastroenterology |  |  |  |
| Royal Devon and Exeter NHS Foundation Trust | | | | | 106 | | |
|  | James | W | Hart | Consultant paediatrician |  | |  |
|  | Amanda |  | Thomas | Senior clinical fellow in gastroenterology |  |  |  |
|  | Ben |  | Hamilton | Specialist registrar in gastroenterology |  |  |  |
|  | Claire |  | Gordon | Senior clinical fellow in gastroenterology |  |  |  |
|  | Desmond |  | Chee | Senior clinical fellow in gastroenterology |  |  |  |
|  | Marian |  | Parkinson | Research administrator |  |  |  |
|  | Helen |  | Gardner Thorpe | Research administrator |  |  |  |
| Shrewsbury and Telford Hospital NHS Trust | | | | | 49 | | |
|  | *Jeff | R | Butterworth | Consultant gastroenterologist |  | |  |
|  | Asima |  | Javed | Specialty registrar in gastroenterology |  |  |  |
|  | Sarah |  | Al-Shakhshir | Specialist registrar in gastroenterology |  |  |  |
|  | Rekha |  | Yadagiri | Specialty doctor in gastroenterology |  |  |  |
|  | Sebrene |  | Maher | Medical student |  |  |  |
| St George's University Hospitals NHS Foundation Trust | | | | | 46 | | |
|  | *Richard | CG | Pollok | Professor of gastroenterology |  | |  |
|  | Tze |  | Ng | Clinical fellow in gastroenterology |  |  |  |
|  | Priscilla |  | Appiahene | Physician associate |  |  |  |
|  | Fiona |  | Donovan | Inflammatory bowel disease nurse specialist |  |  |  |
|  | James |  | Lok | Specialist registrar in gastroenterology |  |  |  |
| St Helens and Knowsley Teaching Hospitals NHS Trust | | | | | 56 | | |
|  | *Rajiv |  | Chandy | Consultant gastroenterologist |  | |  |
|  | *Reema |  | Jagdish | Specialty doctor in gastroenterology |  |  |  |
|  | Daniyal |  | Baig | Specialist registrar in gastroenterology |  |  |  |
| Stockport NHS Foundation Trust | | | | | 15 | | |
|  | *Zahid |  | Mahmood | Consultant gastroenterologist |  | |  |
|  | Liane |  | Marsh | Clinical research practitioner |  |  |  |
| Taunton and Somerset NHS Foundation Trust | | | | | 22 | | |
|  | *Alison |  | Moss | Research nurse |  | |  |
|  | Amin |  | Abdulgader | Clinical fellow in gastroenterology |  |  |  |
|  | Angus |  | Kitchin | Clinical fellow in gastroenterology |  |  |  |
| Torbay and South Devon NHS Foundation Trust | | | | | 39 | | |
|  | *Gareth | J | Walker | Consultant gastroenterologist |  | |  |
|  | Becky |  | George | Inflammatory bowel disease clinical nurse specialist |  |  |  |
|  | Yuen-Hui |  | Lim | Specialist registrar in gastroenterology |  |  |  |
|  | James |  | Gulliver | Specialist registrar in gastroenterology |  |  |  |
| University College London Hospitals NHS Foundation Trust | | | | | 157 | | |
|  | *Stuart |  | Bloom | Professor of gastroenterology |  | |  |
|  | Holly |  | Theaker | Clinical fellow in gastroenterology |  |  |  |
|  | Sean |  | Carlson | Internal medical trainee |  |  |  |
| University Hospital Southampton NHS Foundation Trust | | | | | 24 | | |
|  | *JR | Fraser | Cummings | Consultant gastroenterologist |  | |  |
|  | Robert |  | Livingstone | Clinical fellow in gastroenterology |  |  |  |
| University Hospitals Bristol and Weston NHS Foundation Trust - Bristol | | | | | 45 | | |
|  | *Amanda |  | Beale | Consultant gastroenterologist |  | |  |
|  | Josiah | O | Carter | Specialist registrar in gastroenterology |  |  |  |
| University Hospitals Bristol and Weston NHS Foundation Trust - Weston | | | | | 14 | | |
|  | *Andrew |  | Bell | Consultant gastroenterologist |  | |  |
|  | Archibald |  | Coulter | Specialist registrar in gastroenterology |  |  |  |
| University Hospitals Dorset NHS Foundation Trust | | | | | 72 | | |
|  | *Jonathon |  | Snook | Consultant gastroenterologist |  | |  |
|  | Helen |  | Stone | Specialist registrar in gastroenterology |  |  |  |

|  |  | **Immunogenicity to first anti-TNF** | |  |
| --- | --- | --- | --- | --- |
| **Variable** | **Level** | **Yes**  (n = 704) | **No**  (n = 142) | **P-value** |
| **Gender** | Male | 82.8% (357/431) | 17.2% (74/431) | 0.783 |
|  | Female | 83.6% (347/415) | 16.4% (68/415) |  |
| **Age (years)** | At first anti-TNF | 28.6 (17.8 - 45.6) | 26.2 (18.1 - 42.7) | 0.432 |
|  | Paediatric  (<18 years old) | 83.9% (182/217) | 16.1% (35/217) | 0.833 |
| **Ethnicity** | White: British | 83.0% (571/688) | 17.0% (117/688) | 0.071 |
|  | Black: Caribbean | 80.0% (4/5) | 20.0% (1/5) |  |
|  | Asian: Indian | 76.2% (16/21) | 23.8% (5/21) |  |
| **Smoking** | Current Smoker | 87.1% (101/116) | 12.9% (15/116) | 0.284 |
| **Disease** | Crohn’s disease | 83.1% (493/593) | 16.9% (100/593) | 0.818 |
|  | Ulcerative colitis | 83.9% (183/218) | 16.1% (35/218) |  |
|  | IBD-U | 80.0% (28/35) | 20.0% (7/35) |  |
| **Location** | L1 | 81.9% (118/144) | 18.1% (26/144) | 0.194 |
|  | L2 | 87.9% (131/149) | 12.1% (18/149) |  |
|  | L3 | 81.9% (240/293) | 18.1% (53/293) |  |
|  | L4 | 66.7% (4/6) | 33.3% (2/6) |  |
| **L4 modifier** | True | 80.2% (105/131) | 19.8% (26/131) | 0.286 |
| **Behaviour** | B1 | 88.2% (321/364) | 11.8% (43/364) | <0.001 |
|  | B2 | 76.2% (93/122) | 23.8% (29/122) |  |
|  | B3 | 73.8% (79/107) | 26.2% (28/107) |  |
| **Perianal disease** | True | 81.1% (163/201) | 18.9% (38/201) | 0.355 |
| **Extent** | E1 | 86.4% (19/22) | 13.6% (3/22) | 0.829 |
|  | E2 | 81.7% (85/104) | 18.3% (19/104) |  |
|  | E3 | 84.3% (107/127) | 15.7% (20/127) |  |
| **First anti-TNF indication** | Luminal disease | 83.0% (679/818) | 17.0% (139/818) | 0.605 |
|  | Extraintestinal | 78.9% (15/19) | 21.1% (4/19) | 0.543 |
|  | Co-existing non-IBD diagnosis | 100.0% (5/5) | 0.0% (0/5) | 0.596 |
| **Immunomodulator** | Start TNF1 | 78.8% (364/462) | 21.2% (98/462) | <0.001 |
| **Immunomodulator type** | Azathioprine | 78.1% (235/301) | 21.9% (66/301) | 0.796 |
|  | Mercaptopurine | 83.8% (62/74) | 16.2% (12/74) |  |
|  | Tioguanine | (0/0) | (0/0) |  |
|  | Methotrexate | 81.1% (43/53) | 18.9% (10/53) |  |
| **Duration (years)** | TNF1 | 1.3 (0.7 - 2.7) | 1.9 (0.8 - 4.3) | 0.001 |
| **Drug level** | TNF1 | 0.7 (0.7 - 3.2) | 4.2 (2.6 - 7.6) | <0.001 |
| **Dosing regimen** | Escalated | 83.0% (338/407) | 17.0% (69/407) | 0.927 |
|  | Standard | 83.4% (366/439) | 16.6% (73/439) |  |
| **Treatment outcome** | Treatment failure | 83.8% (550/656) | 16.2% (106/656) | 0.619 |
|  | Adverse event | 81.4% (48/59) | 18.6% (11/59) |  |
|  | Non-treatment failure | 80.9% (106/131) | 19.1% (25/131) |  |

### **Supplemental Table 2:** Variables associated with development of immunogenicity to first anti-TNF, stratified by biologic drug (infliximab as first anti-TNF)

###

|  |  | **Immunogenicity to first anti-TNF** | |  |
| --- | --- | --- | --- | --- |
| **Variable** | **Level** | **Yes**  (n = 99) | **No**  (n = 113) | **P-value** |
| **Gender** | Male | 50.5% (51/101) | 49.5% (50/101) | 0.335 |
|  | Female | 43.2% (48/111) | 56.8% (63/111) |  |
| **Age (years)** | At first anti-TNF | 33.8 (22.7 - 47.3) | 32.9 (24.1 - 43.6) | 0.894 |
|  | Pediatric  (<18 years old) | 65.0% (13/20) | 35.0% (7/20) | 0.102 |
| **Ethnicity** | White: British | 42.5% (76/179) | 57.5% (103/179) | 0.059 |
|  | Black: Caribbean | 0.0% (0/1) | 100.0% (1/1) |  |
|  | Asian: Indian | 75.0% (3/4) | 25.0% (1/4) |  |
| **Smoking** | Current | 70.3% (26/37) | 29.7% (11/37) | 0.002 |
| **Weight (kg)** | Start TNF1 | 77.6 (66.0 - 90.5) | 72.0 (63.8 - 87.0) | 0.164 |
| **Disease** | Crohn’s disease | 48.8% (79/162) | 51.2% (83/162) | 0.435 |
|  | Ulcerative colitis | 42.5% (17/40) | 57.5% (23/40) |  |
|  | IBD-U | 30.0% (3/10) | 70.0% (7/10) |  |
| **Location** | L1 | 45.1% (23/51) | 54.9% (28/51) | 0.643 |
|  | L2 | 45.0% (18/40) | 55.0% (22/40) |  |
|  | L3 | 54.4% (37/68) | 45.6% (31/68) |  |
|  | L4 | 33.3% (1/3) | 66.7% (2/3) |  |
| **L4 modifier** | True | 50.0% (16/32) | 50.0% (16/32) | 1.000 |
| **Behaviour** | B1 | 53.8% (49/91) | 46.2% (42/91) | 0.350 |
|  | B2 | 44.1% (15/34) | 55.9% (19/34) |  |
|  | B3 | 59.5% (22/37) | 40.5% (15/37) |  |
| **Perianal disease** | True | 48.5% (16/33) | 51.5% (17/33) | 1.000 |
| **Extent** | E1 | 50.0% (2/4) | 50.0% (2/4) | 0.501 |
|  | E2 | 32.0% (8/25) | 68.0% (17/25) |  |
|  | E3 | 47.6% (10/21) | 52.4% (11/21) |  |
| **First anti-TNF indication** | Luminal disease | 45.8% (92/201) | 54.2% (109/201) | 0.354 |
|  | Extraintestinal | 75.0% (9/12) | 25.0% (3/12) | 0.070 |
|  | Co-existing non-IBD diagnosis | 38.5% (5/13) | 61.5% (8/13) | 0.580 |
| **Immunomodulator** | Start TNF1 | 42.9% (36/84) | 57.1% (48/84) | 0.402 |
| **Immunomodulator type** | Azathioprine | 37.3% (22/59) | 62.7% (37/59) | 0.339 |
|  | Mercaptopurine | 50.0% (7/14) | 50.0% (7/14) |  |
|  | Tioguanine | (0/0) | (0/0) |  |
|  | Methotrexate | 60.0% (6/10) | 40.0% (4/10) |  |
| **Duration (years)** | TNF1 | 1.4 (0.7 - 2.5) | 1.2 (0.6 - 2.7) | 0.455 |
| **Drug level** | TNF1 | 1.1 (0.7 - 3.9) | 9.0 (6.5 - 11.9) | <0.001 |
| **Dosing regimen** | Escalated | 42.3% (33/78) | 57.7% (45/78) | 0.392 |
|  | Standard | 49.3% (66/134) | 50.7% (68/134) |  |
| **Treatment outcome** | Treatment failure | 47.9% (91/190) | 52.1% (99/190) | 0.015 |
|  | Adverse event | 9.1% (1/11) | 90.9% (10/11) |  |
|  | Non-treatment failure | 63.6% (7/11) | 36.4% (4/11) |  |

### **Supplemental Table 3:** Variables associated with development of immunogenicity to first anti-TNF, stratified by biologic drug (adalimumab as first anti-TNF)

| Infliximab as first anti-TNF | | | | |
| --- | --- | --- | --- | --- |
| Treatment failure to first anti-TNF | | **Immunogenic - pharmacokinetic**  Antibody present, low drug level  (n = 414) | **Immunogenic - pharmacodynamic**  Antibody present, adequate drug level  (n = 136) | **P-value** |
| Antibody concentration (AU/mL) (IQR) | | 102.0 (42.0 – 336.8) | 45.5 (16.0 – 72.8) | <0.001 |
| Antibody concentration quartiles | 1 | 2.7% (11/414) | 5.9% (8/136) | <0.001 |
|  | 2 | 26.1% (108/414) | 52.2% (71/136) |  |
|  | 3 | 36.2% (150/414) | 31.6% (43/136) |  |
|  | 4 | 35.0% (145/414) | 10.3% 9 (14/136) |  |
| Drug level (mg/L) (IQR) | | 0.7 (0.7 – 0.7) | 5.5 (4.0 – 9.1) | <0.001 |
| Drug level quartiles (mg/L) | 1 | 43.2% (179/414) | 0 (0/136) | <0.001 |
|  | 2 | 40.3% (167/414) | 0 (0/136) |  |
|  | 3 | 16.4% (68/414) | 48.5% (66/136) |  |
|  | 4 | 0 (0/414) | 51.5% (70/136) |  |
| Adalimumab as first anti-TNF | | | | |
| Treatment failure to first anti-TNF | | **Immunogenic - pharmacokinetic**  Antibody present, low drug level  (n = 73) | **Immunogenic - pharmacodynamic**  Antibody present, adequate drug level  (n = 18) | **P-value** |
| Antibody concentration (AU/mL) (IQR) | | 201.0 (98.0 – 201.0) | 15.5 (11.2 – 62.8) | <0.001 |
| Antibody concentration (AU/mL) quartiles | 1 | 1.4% (1/73) | 11.1% (2/18) | <0.001 |
|  | 2 | 15.1% (11/73) | 61.1% (11/18) |  |
|  | 3 | 23.3% (17/73) | 22.2% (4/18) |  |
|  | 4 | 60.3% (44/73) | 5.6% (1/18) |  |
| Drug level (mg/L) (IQR) | | 0.7 (0.7 – 1.8) | 6.5 (5.9 – 11.4) | <0.001 |
| Drug level quartiles (mg/L) | 1 | 35.6% (26/73) | 0 (0/18) | <0.001 |
|  | 2 | 43.8% (32/73) | 0 (0/18) |  |
|  | 3 | 20.5% (15/73) | 5.6% (1/18) |  |
|  | 4 | 0 (0/73) | 94.4% (17/18) |  |

### **Supplemental Table 4:** Antibody concentration and drug level profiles, of patients who developed immunogenic-pharmacokinetic and immunogenic-pharmacodynamic failure to first anti-TNF, stratified by anti-TNF

|  | **Crohn’s disease** | |
| --- | --- | --- |
|  | Infliximab-first, adalimumab-second  (n = 593) | Adalimumab-first,  Infliximab-second  (n = 162) |
| Risk of developing immunogenicity | OR 2.52  (95% CI 1.43 - 4.68) | OR 2.94  (95% CI 1.48 – 5.94) |
| Risk of developing immunogenic-pharmacokinetic failure (undetectable drug level) | OR 2.61  (95% CI 1.35 – 5.27) | OR 1.65  (95% CI 0.70 – 3.78) |
|  | **Ulcerative colitis** | |
|  | Infliximab-first, adalimumab-second  (n = 218) | Adalimumab-first,  Infliximab-second  (n = 40) |
| Risk of developing immunogenicity | OR 1.16  (95% CI 0.50 – 2.89) | OR 0.85  (95% CI 0.18 – 3.71) |
| Risk of developing immunogenic-pharmacokinetic failure (undetectable drug level) | OR 2.26  (95% CI 0.80 – 7.34) | OR 4.14  (95% CI 0.59 – 30.06) |

### **Supplemental Table 5:** Risk of developing immunogenicity and immunogenic-pharmacokinetic failure (undetectable drug level) to second anti-TNF, stratified by condition

|  | **First anti-TNF** | | **Second anti-TNF** | | **Total** |
| --- | --- | --- | --- | --- | --- |
|  | **Adalimumab**  (n = 12) | **Infliximab**  (n = 58) | **Adalimumab**  (n = 33) | **Infliximab** (n = 12) |  |
| **Adverse event** | | | | |  |
| Infusion reaction | 0.0% (0/12) | 69.0% (40/58) | 0.0% (0/33) | 66.7% (8/12) | 41.7% (48/115) |
| Psoriasiform dermatitis | 0.0% (0/12) | 8.6% (5/58) | 24.2% (8/33) | 8.3% (1/12) | 12.2% (14/115) |
| Rash (not otherwise specified) | 25.0% (3/12) | 6.9% (4/58) | 15.2% (5/33) | 8.3% (1/12) | 11.3% (13/115) |
| Injection site reaction | 50.0% (6/12) | 0.0% (0/58) | 18.2% (6/33) | 0.0% (0/12) | 10.4% (12/115) |
| Arthritis | 16.7% (2/12) | 0.0% (0/58) | 3.0% (1/33) | 8.3% (1/12) | 3.5% (4/115) |
| Viral infection | 0.0% (0/12) | 1.7% (1/58) | 9.1% (3/33) | 0.0% (0/12) | 3.5% (4/115) |
| Deranged liver function tests | 0.0% (0/12) | 3.4% (2/58) | 3.0% (1/33) | 0.0% (0/12) | 2.6% (3/115) |
| Lupus-like syndrome | 0.0% (0/12) | 1.7% (1/58) | 3.0% (1/33) | 8.3% (1/12) | 2.6% (3/115) |
| Headache | 0.0% (0/12) | 1.7% (1/58) | 3.0% (1/33) | 0.0% (0/12) | 1.7% (2/115) |
| Leukocytoclastic vasculitis | 0.0% (0/12) | 1.7% (1/58) | 3.0% (1/33) | 0.0% (0/12) | 1.7% (2/115) |
| Clinically isolated syndrome | 0.0% (0/12) | 0.0% (0/58) | 3.0% (1/33) | 0.0% (0/12) | 0.9% (1/115) |
| Deranged renal function | 0.0% (0/12) | 0.0% (0/58) | 3.0% (1/33) | 0.0% (0/12) | 0.9% (1/115) |
| Interstitial lung disease | 0.0% (0/12) | 0.0% (0/58) | 3.0% (1/33) | 0.0% (0/12) | 0.9% (1/115) |
| Night sweats | 0.0% (0/12) | 1.7% (1/58) | 0.0% (0/33) | 0.0% (0/12) | 0.9% (1/115) |
| Oral granulomatosis | 8.3% (1/12) | 0.0% (0/58) | 0.0% (0/33) | 0.0% (0/12) | 0.9% (1/115) |
| Peripheral neuropathy | 0.0% (0/12) | 0.0% (0/58) | 3.0% (1/33) | 0.0% (0/12) | 0.9% (1/115) |
| Small bowel adenocarcinoma | 0.0% (0/12) | 0.0% (0/58) | 3.0% (1/33) | 0.0% (0/12) | 0.9% (1/115) |
| Systemic inflammatory response syndrome | 0.0% (0/12) | 1.7% (1/58) | 0.0% (0/33) | 0.0% (0/12) | 0.9% (1/115) |
| Tuberculosis (miliary) | 0.0% (0/12) | 0.0% (0/58) | 3.0% (1/33) | 0.0% (0/12) | 0.9% (1/115) |
| Vasculitis | 0.0% (0/12) | 1.7% (1/58) | 0.0% (0/33) | 0.0% (0/12) | 0.9% (1/115) |
| **Serious** | | | | |  |
| Yes | 0.0% (0/12) | 5.2% (3/58) | 24.2% (8/33) | 0.0% (0/12) | 9.6% (11/115) |
| No | 100.0% (12/12) | 94.8% (55/58) | 75.8% (25/33) | 100.0% (12/12) | 90.4% (104/115) |
| **Severity** | | | | |  |
| Mild | 33.3% (4/12) | 36.2% (21/58) | 36.4% (12/33) | 33.3% (4/12) | 35.7% (41/115) |
| Moderate | 66.7% (8/12) | 63.8% (37/58) | 57.6% (19/33) | 66.7% (8/12) | 62.6% (72/115) |
| Severe | 0.0% (0/12) | 0.0% (0/58) | 6.1% (2/33) | 0.0% (0/12) | 1.7% (2/115) |
| **Causality** | | | | |  |
| Not related | 0.0% (0/12) | 0.0% (0/58) | 0/0% (0/33) | 0.0% (0/12) | 0.0% (0/115) |
| Unlikely | 8.3% (1/12) | 0.0% (0/58) | 6.1% (2/33) | 8.3% (1/12) | 3.5% (4/115) |
| Possibly | 16.7% (2/12) | 17.2% (10/58) | 33.3% (11/33) | 8.3% (1/12) | 20.9% (24/115) |
| Probably | 25.0% (3/12) | 37.9% (22/58) | 39.4% (13/33) | 41.7% (5/12) | 37.4% (43/115) |
| Definitely | 50.0% (6/12) | 44.8% (26/58) | 21.2% (7/33) | 41.7% (5/12) | 38.3% (44/115) |

### **Supplemental Table 6:** Adverse Events leading to drug cessation

| **Therapy** | **Number of patients**  (n = 1058) |
| --- | --- |
| Anti-TNF therapy  Infliximab  Adalimumab  Certolizumab  Golimumab | 16 (1.5)  6 (0.6)  2 (0.2)  2 (0.2)  6 (0.6) |
| Vedolizumab | 173 (16.4) |
| Ustekinumab | 198 (18.7) |
| Tofacitinib | 16 (1.5) |
| 5 ASA-monotherapy | 5 (0.5) |
| Thiopurine monotherapy | 15 (1.4) |
| Methotrexate monotherapy | 7 (0.7) |
| Exclusive enteral nutrition | 2 (0.2) |
| Long-term corticosteroids | 4 (0.4) |
| Ciclosporin | 0 (0) |
| Metronidazole | 1 (0.1) |
| Thalidomide and sirolimus | 1 (0.1) |
| Cannabidiol oil | 1 (0.1) |
| Clinical trial | 1 (0.1) |
| Surgery | 32 (3.0) |
| No treatment | 34 (3.2) |
| Remained on second anti-TNF | 552 (52.2) |

### **Supplemental Table 7:** Therapies following cessation to second anti-TNF

### **
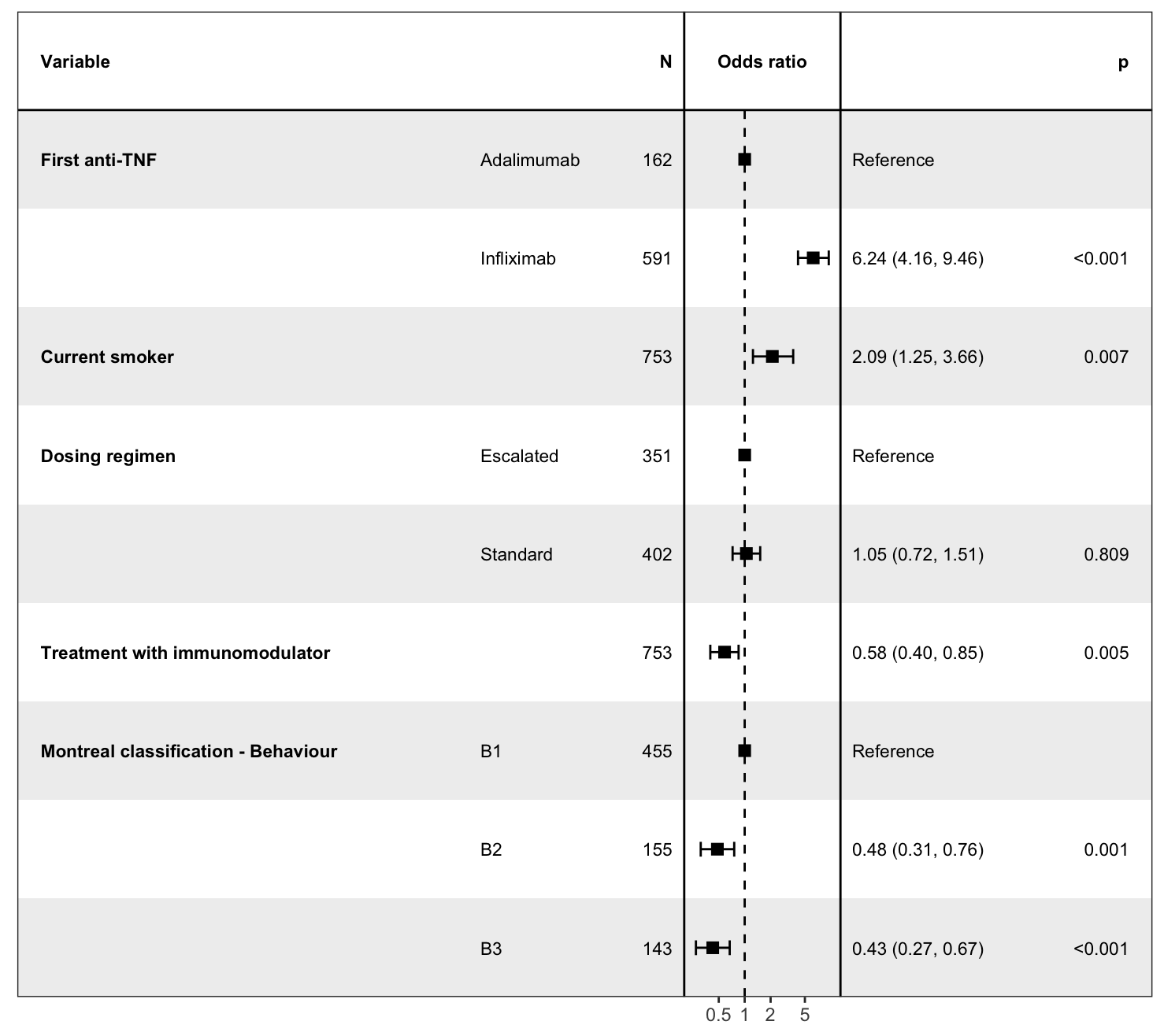
Supplemental Figure 1:** Forest plot showing the coefficients from a multivariable logistic regression model of associations with immunogenicity first anti-TNF, stratified by condition (Crohn’s disease)

###
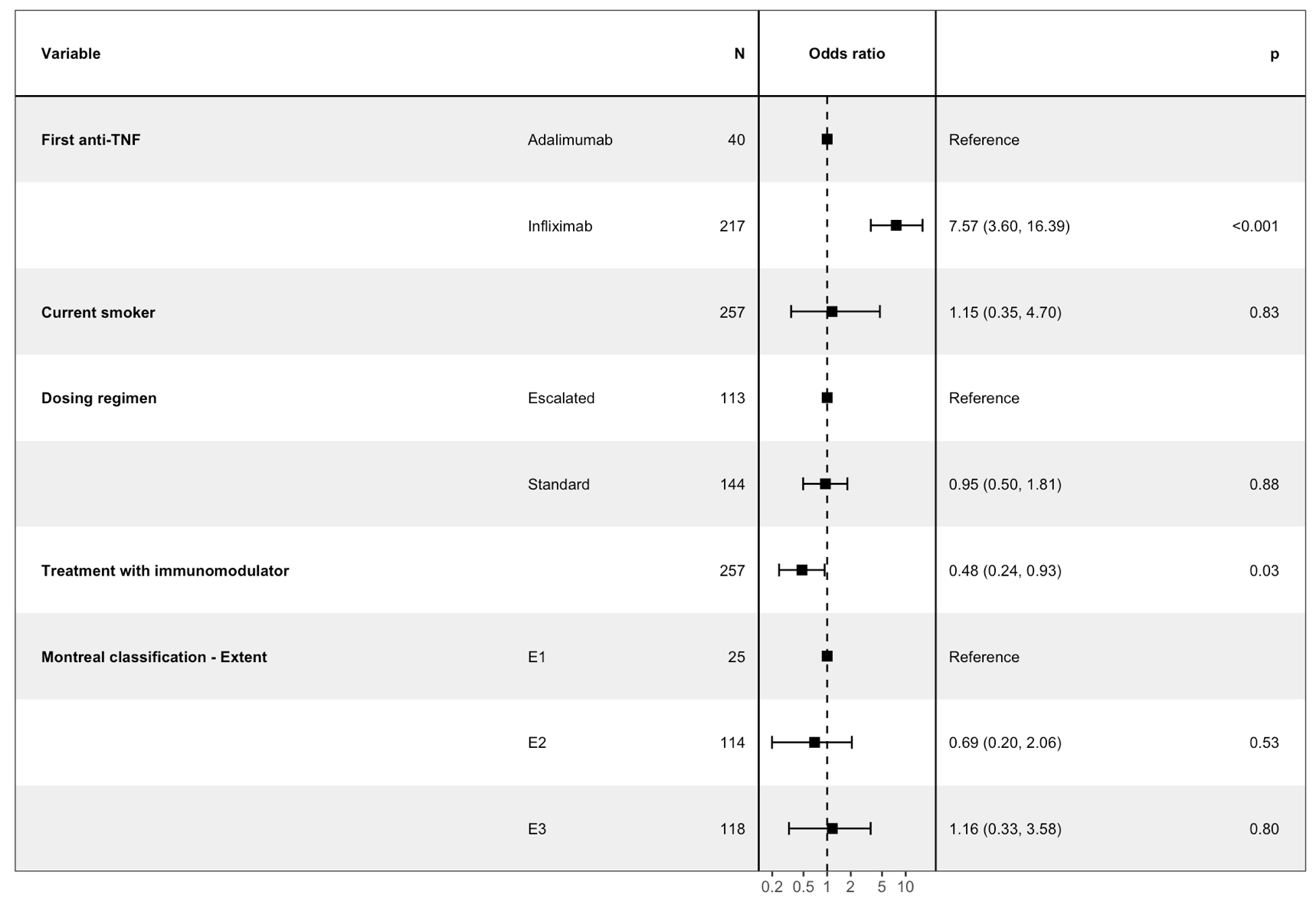
**Supplemental Figure 2:** Forest plot showing the coefficients from a multivariable logistic regression model of associations with immunogenicity first anti-TNF, stratified by condition (ulcerative colitis)

###
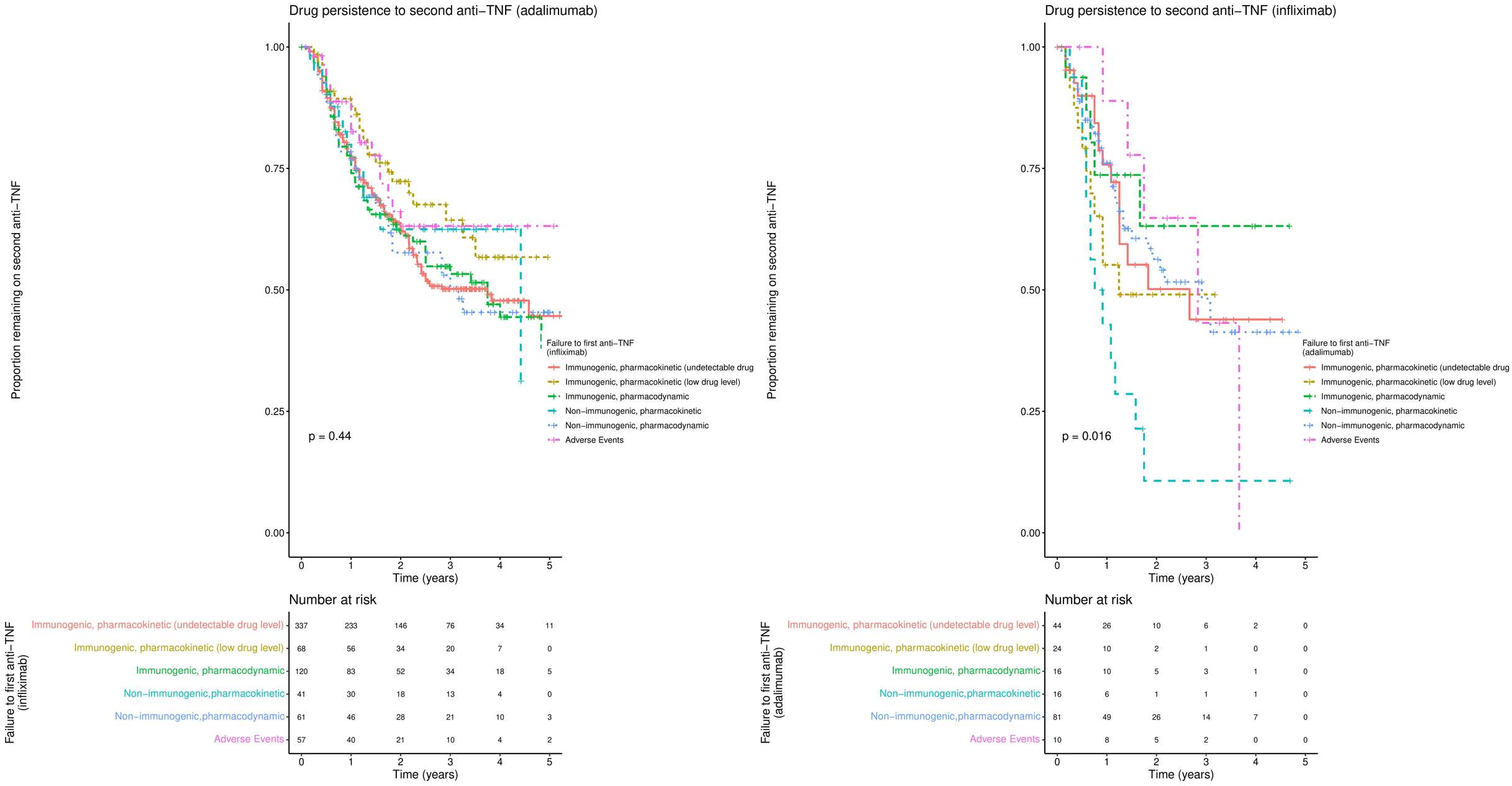
**Supplemental Figure 3:** Drug persistence to second anti-TNF, including stricter definition of immunogenic, pharmacokinetic failure (antibody present, undetectable drug level), stratified by first anti-TNF and type of failure to first anti-TNF

###
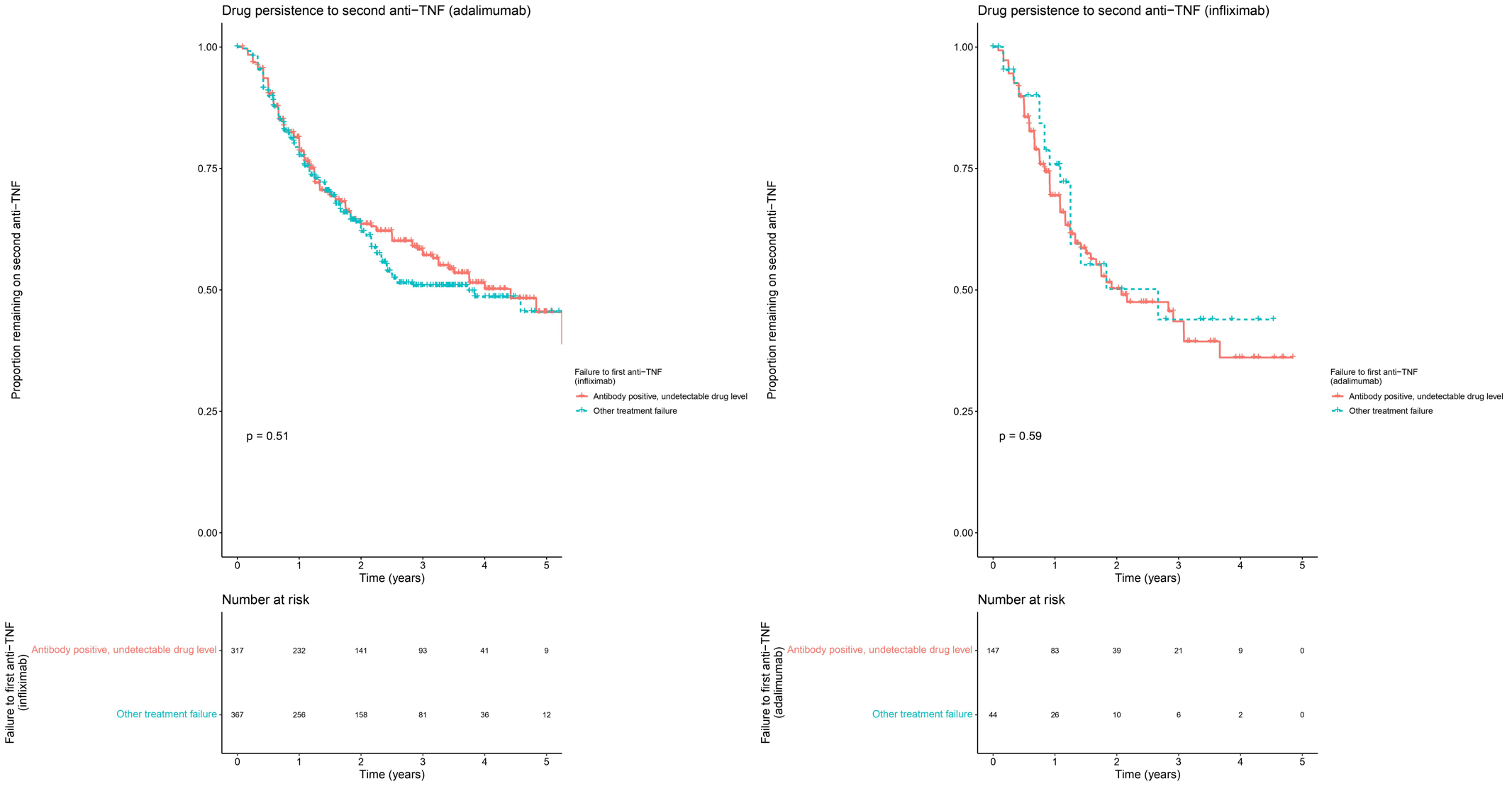
**Supplemental Figure 4:** Drug persistence to second anti-TNF, comparing patients who developed immunogenic-pharmacokinetic failure (antibody present, undetectable drug level) to patients who developed treatment failure for other reasons
